# Crystal structure of piperazine-1,4-diium bis­(4-amino­benzene­sulfonate)

**DOI:** 10.1107/S2056989015024457

**Published:** 2015-12-31

**Authors:** K. Sathesh Kumar, S. Ranjith, S. Sudhakar, P. Srinivasan, M. N. Ponnuswamy

**Affiliations:** a Department of Physics, SRM University, Ramapuram Campus, Chennai 600 089, India; b Department of Physics, Alagappa University, Karaikudi 630 003, India; c Department of Physics, University College of Engineering, Panruti, Cuddalore 607 106, India; d Centre of Advanced Study in Crystallography and Biophysics, University of Madras, Guindy Campus, Chennai 600 025, India

**Keywords:** crystal structure, piperazine, 4-amino­benzene­sulfonate, hydrogen bonding, three-dimensional framework

## Abstract

The asymmetric unit of the title salt, C_4_H_12_N_2_^2+^·2C_6_H_6_NO_3_S^−^, consists of half a piperazindiium dication, located about an inversion centre, and a 4-amino­benzene­sulfonate anion. The piperazine ring adopts a chair conformation. In the crystal, the cations and anions are linked *via* N—H⋯O and C—H⋯O hydrogen bonds, forming a three-dimensional framework. Within the framework there are C—H⋯π inter­actions and the N—H⋯O hydrogen bonds result in the formation of *R*_4_^4^(22) and *R*_3_^4^(13) ring motifs.

## Related literature   

For examples of the the numerous biological activities of piperazines and their various salts, see: Kaur *et al.* (2010 ▸); Eswaran *et al.* (2010 ▸); Chou *et al.* (2010 ▸); Chen *et al.* (2004 ▸); Shingalapur *et al.* (2009 ▸); Shchekotikhin *et al.* (2005 ▸); Faist *et al.* (2012 ▸); Kulig *et al.* (2007 ▸). For a related structure, see: Wei (2011 ▸). 
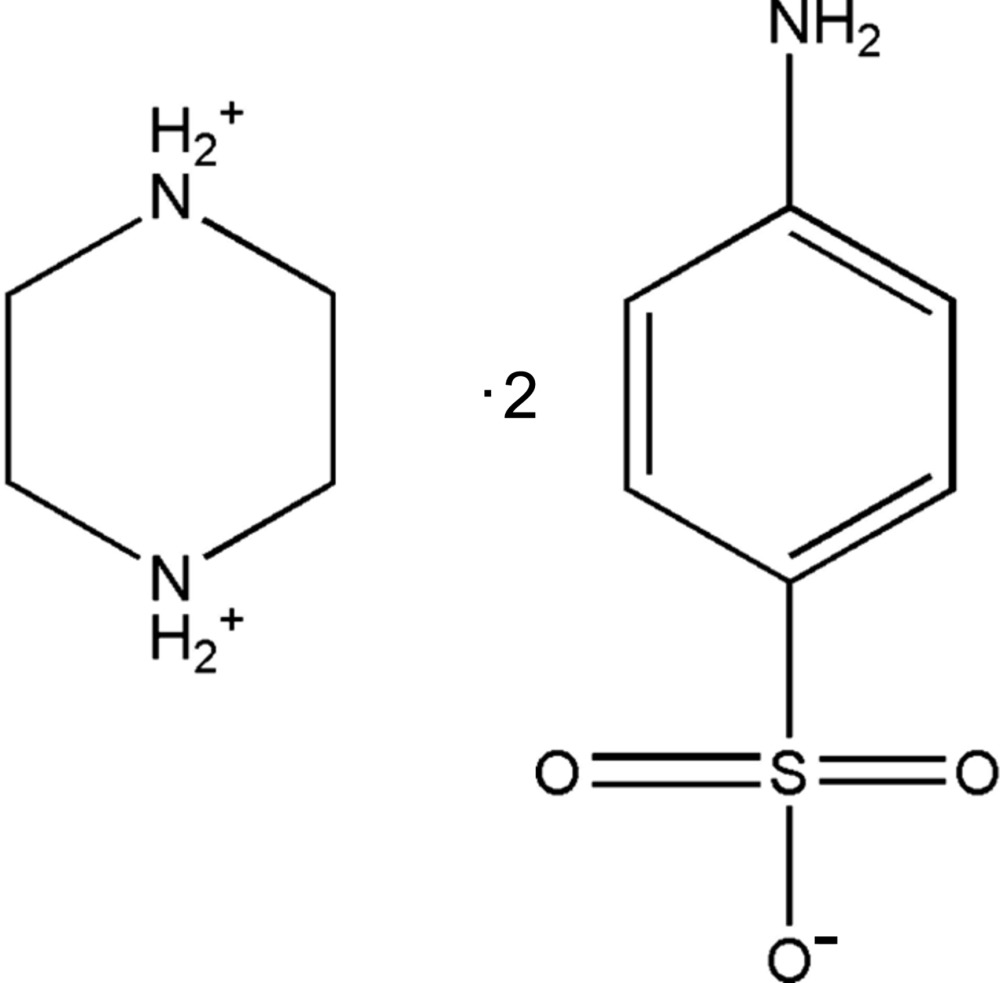


## Experimental   

### Crystal data  

C_4_H_12_N_2_^2+^·2C_6_H_6_NO_3_S^−^
*M* *_r_* = 432.52
Orthorhombic, 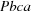

*a* = 10.1709 (4) Å
*b* = 8.4461 (3) Å
*c* = 21.5569 (9) Å
*V* = 1851.83 (12) Å^3^
*Z* = 4
Mo *K*α radiation
μ = 0.33 mm^−1^
*T* = 293 K
0.25 × 0.22 × 0.19 mm

### Data collection  

Bruker APEXII CCD area-detector diffractometer
Absorption correction: multi-scan (*SADABS*; Bruker, 2008 ▸) *T*_min_ = 0.920, *T*_max_ = 0.939
31521 measured reflections
2731 independent reflections
2130 reflections with *I* > 2σ(*I*)
*R*_int_ = 0.039

### Refinement  

*R*[*F* ^2^ > 2σ(*F* ^2^)] = 0.038
*wR*(*F* ^2^) = 0.107
*S* = 1.03
2731 reflections
160 parameters
H atoms treated by a mixture of independent and constrained refinement
Δρ_max_ = 0.73 e Å^−3^
Δρ_min_ = −0.42 e Å^−3^

### 

Data collection: *APEX2* (Bruker, 2008 ▸); cell refinement: *SAINT* (Bruker, 2008 ▸); data reduction: *SAINT*; program(s) used to solve structure: *SHELXS97* (Sheldrick, 2008 ▸); program(s) used to refine structure: *SHELXL97* (Sheldrick, 2008 ▸); molecular graphics: *ORTEP-3 for Windows* (Farrugia, 2012 ▸) and *Mercury* (Macrae *et al.*, 2008 ▸); software used to prepare material for publication: *SHELXL97* and *PLATON* (Spek, 2009 ▸).

## Supplementary Material

Crystal structure: contains datablock(s) global, I. DOI: 10.1107/S2056989015024457/su5262sup1.cif

Structure factors: contains datablock(s) I. DOI: 10.1107/S2056989015024457/su5262Isup2.hkl

**Fig. 1.**
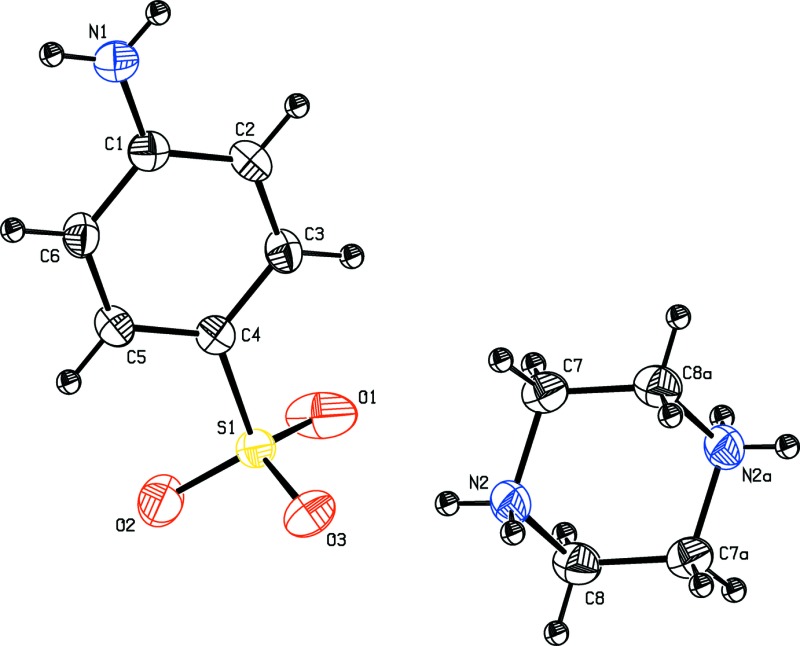
The molecular structure of the title salt, with atom labelling. Displacement ellipsoids are drawn at the 30% probability level. The unlabelled atoms of the cation are related to the labelled atoms by inversion symmetry (- x + 2, − y, − z + 1).

**Fig. 2.**
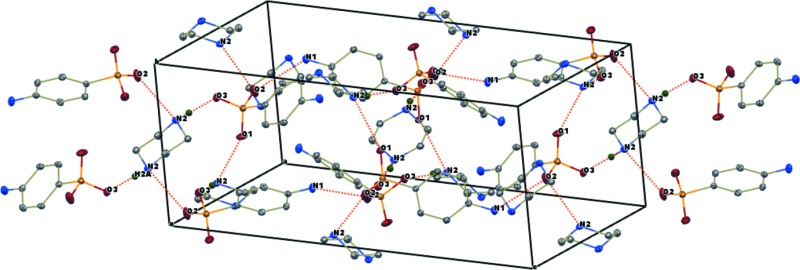
A partial view of the crystal packing of the title salt, viewed along the a axis. Hydrogen-bonded chains (dashed lines) run along the a and c axes (see Table 1).

**Fig. 3.**
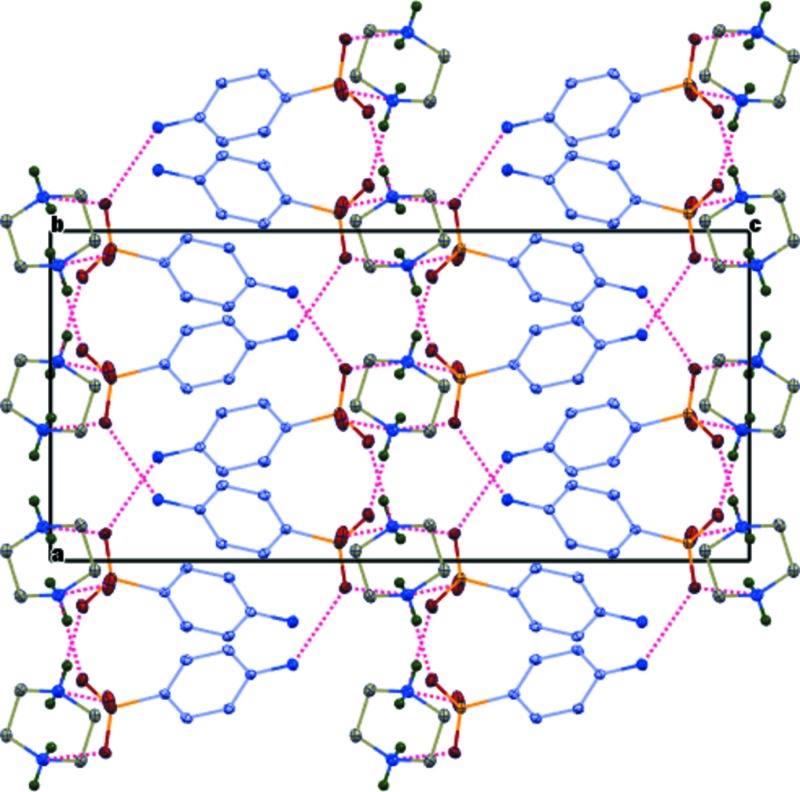
Crystal packing of the title salt, viewed along the b axis, illustrating the formation of the hydrogen-bonded (dashed lines) molecular ribbons running along the b axis direction (see Table 1). For the sake of clarity, H atoms not involved in hydrogen bonds have been omitted.

**Fig. 4.**
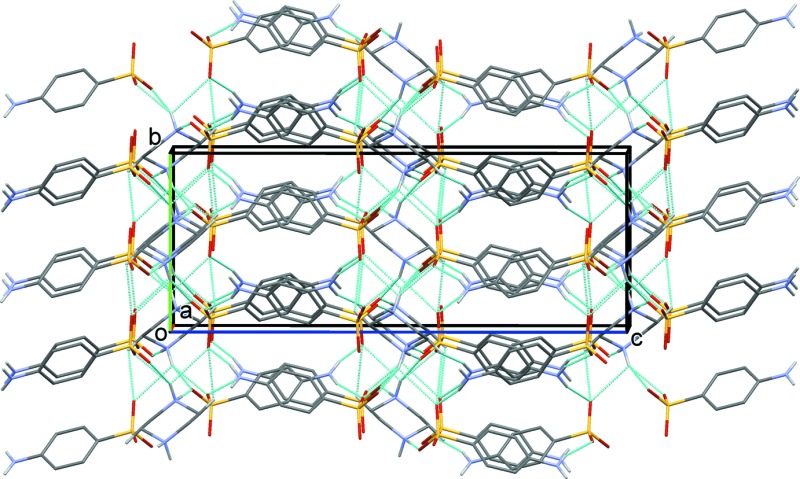
A view along the a axis of the crystal packing of the title salt. The hydrogen bonds are shown as dashed lines (Table 1), and H atoms not involved in these interactions have been omitted for clarity.

Supporting information file. DOI: 10.1107/S2056989015024457/su5262Isup3.cml

x y z . DOI: 10.1107/S2056989015024457/su5262fig1.tif
The mol­ecular structure of the title salt, with atom labelling. Displacement ellipsoids are drawn at the 30% probability level. The unlabelled atoms of the cation are related to the labelled atoms by inversion symmetry (- *x* + 2, − *y*, − *z* + 1).

a a c . DOI: 10.1107/S2056989015024457/su5262fig2.tif
A partial view of the crystal packing of the title salt, viewed along the *a* axis. Hydrogen-bonded chains (dashed lines) run along the *a* and *c* axes (see Table 1).

b b . DOI: 10.1107/S2056989015024457/su5262fig3.tif
Crystal packing of the title salt, viewed along the *b* axis, illustrating the formation of the hydrogen-bonded (dashed lines) mol­ecular ribbons running along the *b* axis direction (see Table 1). For the sake of clarity, H atoms not involved in hydrogen bonds have been omitted.

a . DOI: 10.1107/S2056989015024457/su5262fig4.tif
A view along the *a* axis of the crystal packing of the title salt. The hydrogen bonds are shown as dashed lines (Table 1), and H atoms not involved in these inter­actions have been omitted for clarity.

CCDC reference: 1443504

Additional supporting information:  crystallographic information; 3D view; checkCIF report

## Figures and Tables

**Table 1:** Hydrogen-bond geometry (Å, °) *Cg*1 is the centroid of the C1–C6 ring.

*D*—H⋯*A*	*D*—H	H⋯*A*	*D*⋯*A*	*D*—H⋯*A*
N1—H1*A*⋯O2^i^	0.82 (3)	2.27 (3)	3.066 (2)	164 (2)
N1—H1*B*⋯O1^ii^	0.86 (3)	2.49 (3)	3.296 (3)	156 (2)
N2—H2*A*⋯O3	0.85 (3)	1.92 (3)	2.764 (2)	175 (2)
N2—H2*B*⋯O2^iii^	0.92 (3)	2.19 (2)	2.928 (2)	137 (2)
N2—H2*B*⋯O3^iii^	0.92 (3)	2.54 (2)	3.328 (2)	145 (2)
C7—H7*A*⋯O1^iv^	0.95 (2)	2.50 (2)	3.167 (2)	128 (2)
C6—H6⋯*Cg*1^ii^	0.93	2.92	3.753 (2)	149

Symmetry codes: (i) 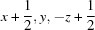; (ii) 
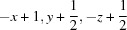
; (iii) 
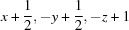
; (iv) 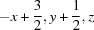.

## supplementary crystallographic information

### S1. Comment

Piperazine derivatives have wide range of applications in pharmaceuticals as anti­malarial (Kaur *et al.*, 2010), anti-tuberculosis (Eswaran *et al.*, 2010), anti­tumor (Chou *et al.*, 2010), anti­cancer (Chen *et al.*, 2004) and anti­viral (Shingalapur *et al.*, 2009) agents. The piperazine nucleus is capable of binding to multiple receptors with high affinity and therefore piperazine has been classified as a privileged structure. In the last decade, a number of piperazine derivatives have been synthesized and evaluated for their cytotoxic activity (Shchekotikhin *et al.*, 2005). The piperazine nucleus has been classified as a privileged structure and is frequently found in biologically active compounds across a number of different therapeutic areas (Faist *et al.*, 2012). Some of these therapeutic areas include anti­microbial, anti-tubercular, anti­convulsant, anti­depressant, anti-inflammatory, cytotoxic, anti­malarial, anti­arrhythmic, anti­oxidant and anti­viral activities *etc*. possessed by the compounds having piperazine nucleus (Kulig *et al.*, 2007). In view of the above said importance,the crystal structure of the title compound has been determined by crystallographic methods.

The molecular structure of the title salt is shown in Fig. 1. The crystallographic inversion centered piperazine ring adopts a chair conformation. The bond lengths N2—C7 and C4—S1 are comparable with the values observed in the related structure piperazine-1,4-diium naphthalene-1,5-di­sulfonate (Wei, 2011). In the anions atom S1 deviates from the benzene ring plane by −0.076 (1) Å. There is a short non-hydrogen contact involving atoms N2···O3 [2.764 Å] at *x*, *y*, *z*.

In the crystal, the N1—H1A···O2 and N1—H1B···O1 hydrogen bonds form an infinite chain leads to the formation of an *R*_4_^4^(22) ring motif (Table 1 and Fig. 2). Similarly, the N2—H2···O hydrogen bonds in the molecular structure results in the formation of an *R*_3_^4^(13) ring motif. These two motifs combine to form a hydrogen-bonded molecular ribbons running along *b* axis (Table 1 and Fig. 3). A C—H···π inter­action is also observed involving atom C6 in the benzene ring of the anion and the centroid of another anion ring with an H···centroid distance of 2.92 Å (Table 1). The molecular structure is stabilized by strong N—H···O hydrogen bonds which form infinite one dimensional chains. These various inter­actions result finally in the formation of a three-dimensional framework structure (Table 1 and Fig. 4).

### S2. Synthesis and crystallization

The title compound was synthesized by slow evaporation at room temperature of an aqueous mixture of piperazine (1.43 g) and sulfanilic acid (2.88 g). Colourless transparent crystals were obtained in a period of 7 days. Single crystals suitable for X-ray diffraction studies were obtained by slow evaporation of a solution in ethyl acetate at room temperature.

### S3. Refinement

Crystal data, data collection and structure refinement details are summarized in Table 2. The NH_2_ and methyl­ene H atoms were located in difference Fourier maps and freely refined. The aromatic CH H atoms were fixed geometrically and treated as riding: C—H = 0.93 Å with *U*_iso_(H) = 1.2*U*_eq_(C)

### Crystal data

**Table:**

C_4_H_12_N_2_^2+^·2C_6_H_6_NO_3_S^−^	*F*(000) = 912
*M**_r_* = 432.52	*D*_x_ = 1.551 Mg m^−^^3^
Orthorhombic, *P**b**c**a*	Mo *K*α radiation, λ = 0.71073 Å
Hall symbol: -P 2ac 2ab	Cell parameters from 2731 reflections
*a* = 10.1709 (4) Å	θ = 2.8–30.8°
*b* = 8.4461 (3) Å	µ = 0.33 mm^−^^1^
*c* = 21.5569 (9) Å	*T* = 293 K
*V* = 1851.83 (12) Å^3^	Block, white crystalline
*Z* = 4	0.25 × 0.22 × 0.19 mm

### Data collection

**Table:**

Bruker APEXII CCD area-detector diffractometer	2731 independent reflections
Radiation source: fine-focus sealed tube	2130 reflections with *I* > 2σ(*I*)
Graphite monochromator	*R*_int_ = 0.039
ω and φ scans	θ_max_ = 30.8°, θ_min_ = 2.8°
Absorption correction: multi-scan (*SADABS*; Bruker, 2008)	*h* = −14→14
*T*_min_ = 0.920, *T*_max_ = 0.939	*k* = −12→10
31521 measured reflections	*l* = −29→30

### Refinement

**Table:**

Refinement on *F*^2^	Secondary atom site location: difference Fourier map
Least-squares matrix: full	Hydrogen site location: inferred from neighbouring sites
*R*[*F*^2^ > 2σ(*F*^2^)] = 0.038	H atoms treated by a mixture of independent and constrained refinement
*wR*(*F*^2^) = 0.107	*w* = 1/[σ^2^(*F*_o_^2^) + (0.0431*P*)^2^ + 1.5618*P*] where *P* = (*F*_o_^2^ + 2*F*_c_^2^)/3
*S* = 1.03	(Δ/σ)_max_ < 0.001
2731 reflections	Δρ_max_ = 0.73 e Å^−^^3^
160 parameters	Δρ_min_ = −0.42 e Å^−^^3^
0 restraints	Extinction correction: *SHELXL97* (Sheldrick, 2008), Fc^*^=kFc[1+0.001xFc^2^λ^3^/sin(2θ)]^-1/4^
Primary atom site location: structure-invariant direct methods	Extinction coefficient: 0.0332 (17)

### Special details

**Table:**

Geometry. All e.s.d.'s (except the e.s.d. in the dihedral angle between two l.s. planes) are estimated using the full covariance matrix. The cell e.s.d.'s are taken into account individually in the estimation of e.s.d.'s in distances, angles and torsion angles; correlations between e.s.d.'s in cell parameters are only used when they are defined by crystal symmetry. An approximate (isotropic) treatment of cell e.s.d.'s is used for estimating e.s.d.'s involving l.s. planes.
Refinement. Refinement of *F*^2^ against ALL reflections. The weighted *R*-factor *wR* and goodness of fit *S* are based on *F*^2^, conventional *R*-factors *R* are based on *F*, with *F* set to zero for negative *F*^2^. The threshold expression of *F*^2^ > σ(*F*^2^) is used only for calculating *R*-factors(gt) *etc*. and is not relevant to the choice of reflections for refinement. *R*-factors based on *F*^2^ are statistically about twice as large as those based on *F*, and *R*- factors based on ALL data will be even larger.

### Fractional atomic coordinates and isotropic or equivalent isotropic displacement parameters (Å^2^)

**Table:**

	*x*	*y*	*z*	*U*_iso_*/*U*_eq_	
C1	0.65542 (16)	0.21272 (19)	0.21371 (7)	0.0266 (3)	
C2	0.72879 (17)	0.09868 (19)	0.24541 (8)	0.0302 (3)	
H2	0.7986	0.0490	0.2255	0.036*	
C3	0.69927 (16)	0.05863 (19)	0.30585 (8)	0.0290 (3)	
H3	0.7489	−0.0179	0.3262	0.035*	
C4	0.59536 (15)	0.13233 (18)	0.33669 (7)	0.0245 (3)	
C5	0.52268 (16)	0.24672 (19)	0.30563 (8)	0.0280 (3)	
H5	0.4540	0.2976	0.3260	0.034*	
C6	0.55133 (16)	0.2859 (2)	0.24479 (8)	0.0292 (3)	
H6	0.5009	0.3615	0.2244	0.035*	
C7	0.97012 (19)	0.0404 (2)	0.43662 (8)	0.0317 (4)	
C8	0.8928 (2)	0.0027 (2)	0.54312 (9)	0.0358 (4)	
N1	0.68534 (18)	0.2535 (2)	0.15357 (7)	0.0380 (4)	
N2	0.89658 (17)	0.11265 (18)	0.48903 (7)	0.0331 (3)	
O1	0.5723 (2)	−0.09171 (16)	0.41653 (7)	0.0586 (5)	
O2	0.41824 (14)	0.1269 (2)	0.42189 (6)	0.0514 (4)	
O3	0.63822 (14)	0.16386 (17)	0.45474 (6)	0.0413 (3)	
S1	0.55342 (4)	0.07560 (5)	0.412784 (18)	0.02600 (14)	
H2B	0.934 (2)	0.207 (3)	0.5007 (11)	0.046 (6)*	
H1A	0.744 (2)	0.202 (3)	0.1367 (11)	0.042 (6)*	
H7A	0.971 (2)	0.119 (3)	0.4049 (11)	0.042 (6)*	
H1B	0.635 (2)	0.318 (3)	0.1336 (11)	0.047 (6)*	
H8B	0.853 (2)	0.057 (3)	0.5758 (11)	0.046 (6)*	
H2A	0.819 (3)	0.132 (3)	0.4765 (11)	0.046 (6)*	
H7B	0.924 (2)	−0.044 (3)	0.4239 (10)	0.033 (5)*	
H8A	0.842 (2)	−0.089 (3)	0.5299 (10)	0.045 (6)*	

### Atomic displacement parameters (Å^2^)

**Table:**

	*U*^11^	*U*^22^	*U*^33^	*U*^12^	*U*^13^	*U*^23^
C1	0.0248 (7)	0.0274 (7)	0.0278 (7)	−0.0045 (6)	−0.0003 (6)	−0.0017 (6)
C2	0.0275 (8)	0.0283 (7)	0.0349 (8)	0.0040 (6)	0.0058 (6)	−0.0030 (6)
C3	0.0274 (8)	0.0261 (7)	0.0336 (8)	0.0045 (6)	−0.0003 (6)	0.0024 (6)
C4	0.0249 (7)	0.0222 (7)	0.0266 (7)	−0.0019 (6)	0.0005 (6)	−0.0004 (6)
C5	0.0258 (7)	0.0261 (7)	0.0319 (8)	0.0030 (6)	0.0040 (6)	0.0009 (6)
C6	0.0258 (8)	0.0300 (8)	0.0318 (8)	0.0030 (6)	−0.0009 (6)	0.0064 (7)
C7	0.0423 (10)	0.0247 (7)	0.0282 (8)	−0.0012 (7)	−0.0005 (7)	−0.0003 (6)
C8	0.0379 (10)	0.0366 (9)	0.0328 (9)	0.0006 (8)	0.0060 (7)	−0.0005 (7)
N1	0.0366 (8)	0.0500 (10)	0.0275 (7)	0.0068 (8)	0.0036 (6)	0.0022 (7)
N2	0.0366 (8)	0.0265 (7)	0.0362 (8)	0.0072 (6)	−0.0033 (7)	−0.0028 (6)
O1	0.1135 (16)	0.0226 (7)	0.0395 (8)	0.0040 (8)	0.0162 (8)	0.0038 (5)
O2	0.0303 (7)	0.0879 (12)	0.0361 (7)	0.0069 (7)	0.0056 (6)	0.0168 (8)
O3	0.0462 (8)	0.0451 (8)	0.0327 (7)	−0.0063 (6)	−0.0078 (6)	−0.0035 (6)
S1	0.0292 (2)	0.0235 (2)	0.0252 (2)	0.00047 (14)	0.00009 (14)	0.00065 (14)

### Geometric parameters (Å, º)

**Table:**

C1—N1	1.376 (2)	C7—H7A	0.95 (2)
C1—C6	1.397 (2)	C7—H7B	0.90 (2)
C1—C2	1.397 (2)	C8—N2	1.491 (2)
C2—C3	1.379 (2)	C8—C7^i^	1.506 (3)
C2—H2	0.9300	C8—H8B	0.93 (2)
C3—C4	1.395 (2)	C8—H8A	0.98 (2)
C3—H3	0.9300	N1—H1A	0.82 (3)
C4—C5	1.389 (2)	N1—H1B	0.86 (3)
C4—S1	1.7614 (16)	N2—H2B	0.92 (3)
C5—C6	1.384 (2)	N2—H2A	0.85 (3)
C5—H5	0.9300	O1—S1	1.4285 (14)
C6—H6	0.9300	O2—S1	1.4548 (15)
C7—N2	1.486 (2)	O3—S1	1.4553 (13)
C7—C8^i^	1.506 (3)	S1—O3	1.4553 (13)
			
N1—C1—C6	120.59 (16)	N2—C8—C7^i^	110.69 (15)
N1—C1—C2	121.04 (16)	N2—C8—H8B	107.1 (15)
C6—C1—C2	118.37 (15)	C7^i^—C8—H8B	107.5 (15)
C3—C2—C1	121.00 (15)	N2—C8—H8A	106.3 (13)
C3—C2—H2	119.5	C7^i^—C8—H8A	112.6 (14)
C1—C2—H2	119.5	H8B—C8—H8A	113 (2)
C2—C3—C4	120.37 (15)	C1—N1—H1A	116.5 (16)
C2—C3—H3	119.8	C1—N1—H1B	119.8 (16)
C4—C3—H3	119.8	H1A—N1—H1B	123 (2)
C5—C4—C3	118.95 (15)	C7—N2—C8	110.61 (14)
C5—C4—S1	120.62 (12)	C7—N2—H2B	111.1 (15)
C3—C4—S1	120.39 (12)	C8—N2—H2B	109.7 (15)
C6—C5—C4	120.74 (15)	C7—N2—H2A	107.8 (16)
C6—C5—H5	119.6	C8—N2—H2A	110.2 (16)
C4—C5—H5	119.6	H2B—N2—H2A	107 (2)
C5—C6—C1	120.56 (15)	O1—S1—O2	114.48 (12)
C5—C6—H6	119.7	O1—S1—O3	113.06 (10)
C1—C6—H6	119.7	O2—S1—O3	108.89 (10)
N2—C7—C8^i^	110.17 (15)	O1—S1—O3	113.06 (10)
N2—C7—H7A	105.4 (14)	O2—S1—O3	108.89 (10)
C8^i^—C7—H7A	111.3 (14)	O1—S1—C4	106.80 (8)
N2—C7—H7B	107.0 (14)	O2—S1—C4	105.87 (8)
C8^i^—C7—H7B	112.6 (14)	O3—S1—C4	107.21 (8)
H7A—C7—H7B	110.0 (19)	O3—S1—C4	107.21 (8)
			
N1—C1—C2—C3	179.55 (16)	O3—O3—S1—O1	0.00 (18)
C6—C1—C2—C3	0.2 (2)	O3—O3—S1—O2	0.00 (18)
C1—C2—C3—C4	−0.3 (3)	O3—O3—S1—C4	0.0 (2)
C2—C3—C4—C5	−0.2 (2)	C5—C4—S1—O1	140.98 (15)
C2—C3—C4—S1	177.43 (13)	C3—C4—S1—O1	−36.62 (17)
C3—C4—C5—C6	0.9 (2)	C5—C4—S1—O2	18.58 (16)
S1—C4—C5—C6	−176.76 (13)	C3—C4—S1—O2	−159.02 (14)
C4—C5—C6—C1	−1.0 (3)	C5—C4—S1—O3	−97.55 (15)
N1—C1—C6—C5	−178.90 (16)	C3—C4—S1—O3	84.85 (15)
C2—C1—C6—C5	0.5 (2)	C5—C4—S1—O3	−97.55 (15)
C8^i^—C7—N2—C8	−57.3 (2)	C3—C4—S1—O3	84.85 (15)
C7^i^—C8—N2—C7	57.6 (2)		

Symmetry code: (i) −*x*+2, −*y*, −*z*+1.

### Hydrogen-bond geometry (Å, º)

Cg1 is the centroid of the C1–C6 ring.

**Table:**

*D*—H···*A*	*D*—H	H···*A*	*D*···*A*	*D*—H···*A*
N1—H1*A*···O2^ii^	0.82 (3)	2.27 (3)	3.066 (2)	164 (2)
N1—H1*B*···O1^iii^	0.86 (3)	2.49 (3)	3.296 (3)	156 (2)
N2—H2*A*···O3	0.85 (3)	1.92 (3)	2.764 (2)	175 (2)
N2—H2*B*···O2^iv^	0.92 (3)	2.19 (2)	2.928 (2)	137 (2)
N2—H2*B*···O3^iv^	0.92 (3)	2.54 (2)	3.328 (2)	145 (2)
C7—H7*A*···O1^v^	0.95 (2)	2.50 (2)	3.167 (2)	128 (2)
C6—H6···*Cg*1^iii^	0.93	2.92	3.753 (2)	149

Symmetry codes: (ii) *x*+1/2, *y*, −*z*+1/2; (iii) −*x*+1, *y*+1/2, −*z*+1/2; (iv) *x*+1/2, −*y*+1/2, −*z*+1; (v) −*x*+3/2, *y*+1/2, *z*.
