# Supplementary figures and images for: Crystal structure of piperazine-1,4-diium bis­(4-amino­benzene­sulfonate)
Source: Acta Crystallogr E Crystallogr Commun. 2015 Dec 31;71(Pt 12):o1084–5. doi: 10.1107/S2056989015024457 (PMC4719991; doi:10.1107/S2056989015024457)

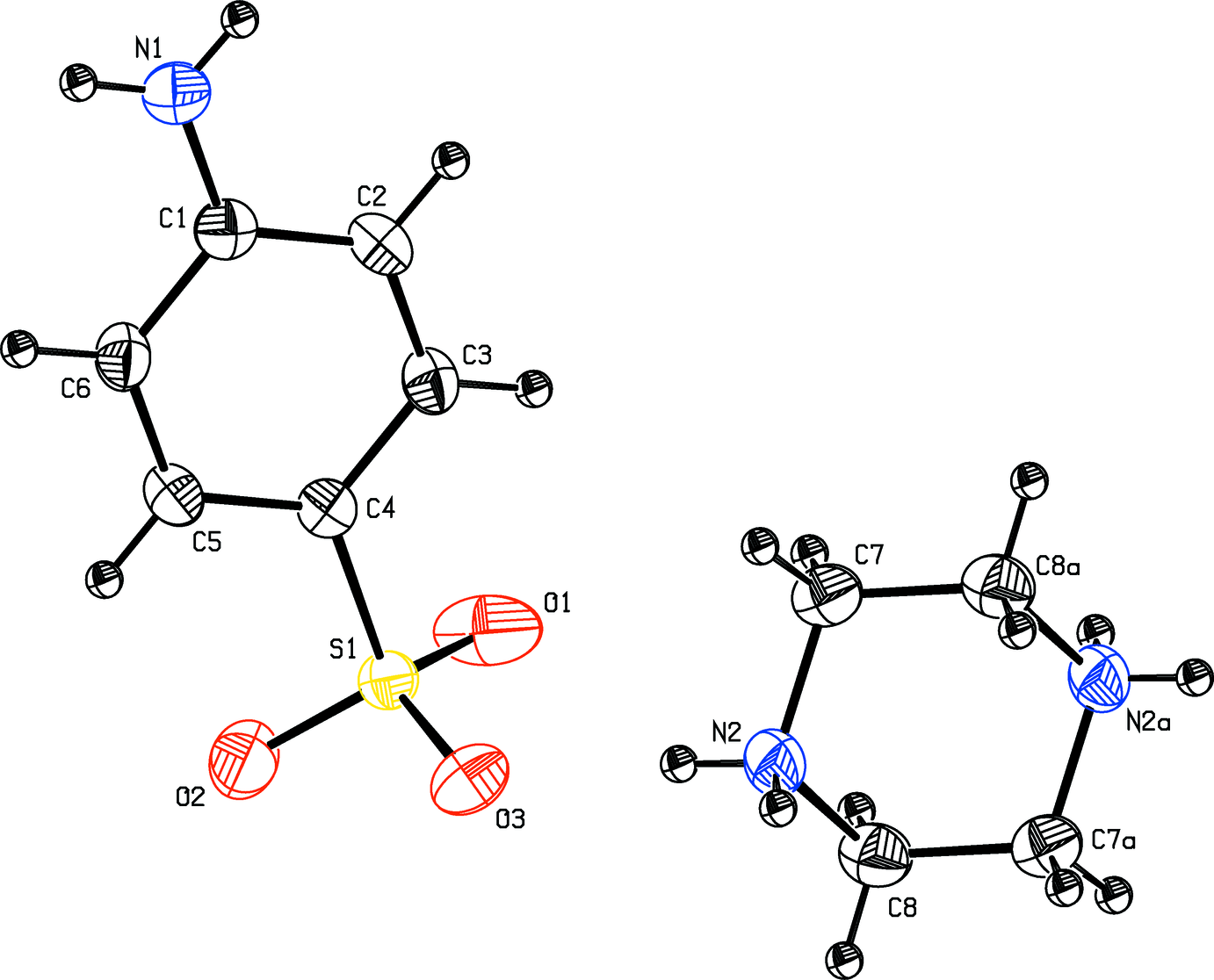

Supplement: Supplementary file 4 [file e-71-o1084-fig1.tif]

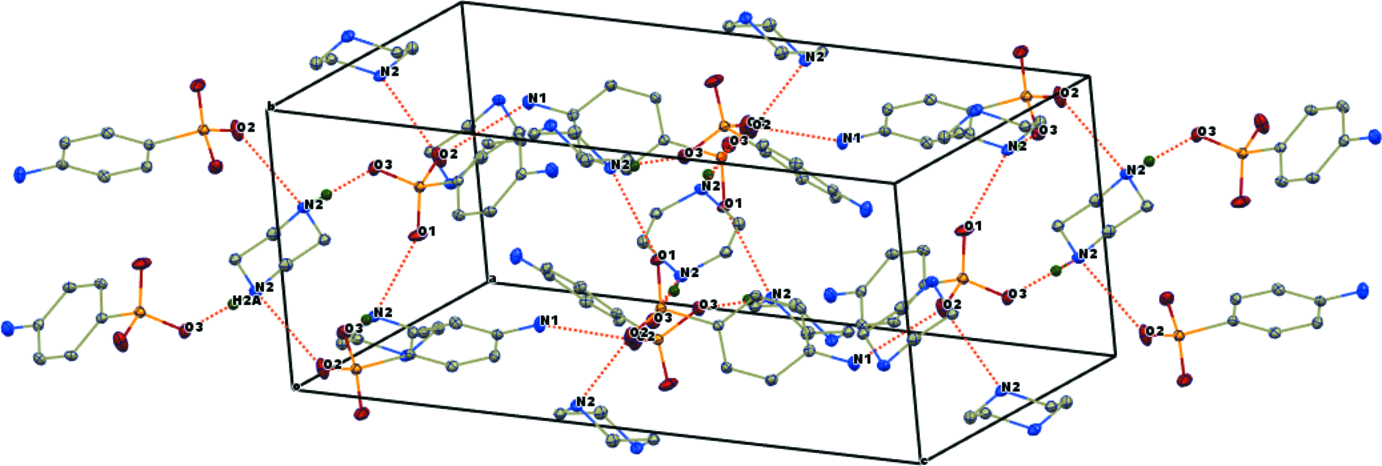

Supplement: Supplementary file 5 [file e-71-o1084-fig2.tif]

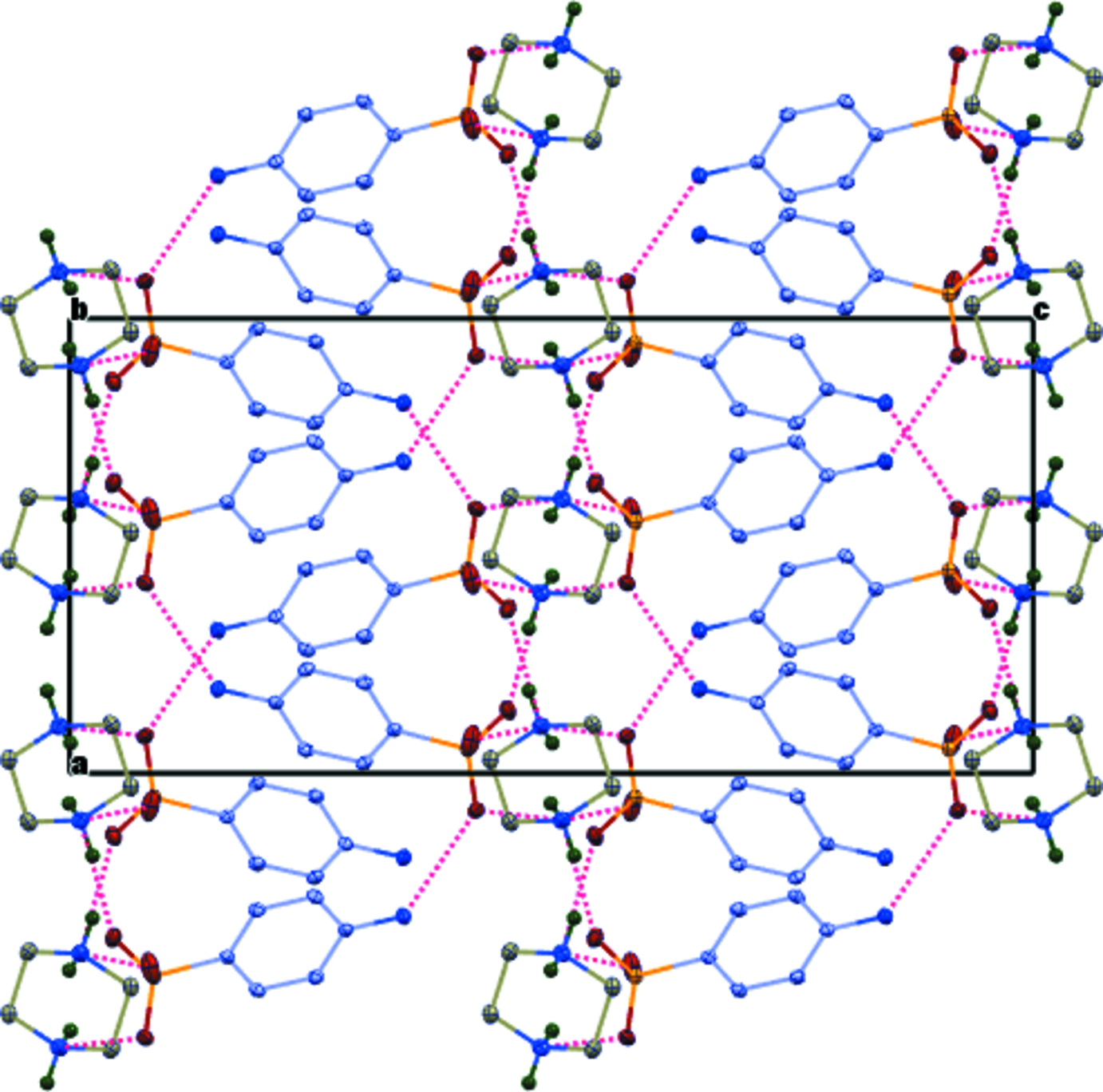

Supplement: Supplementary file 6 [file e-71-o1084-fig3.tif]

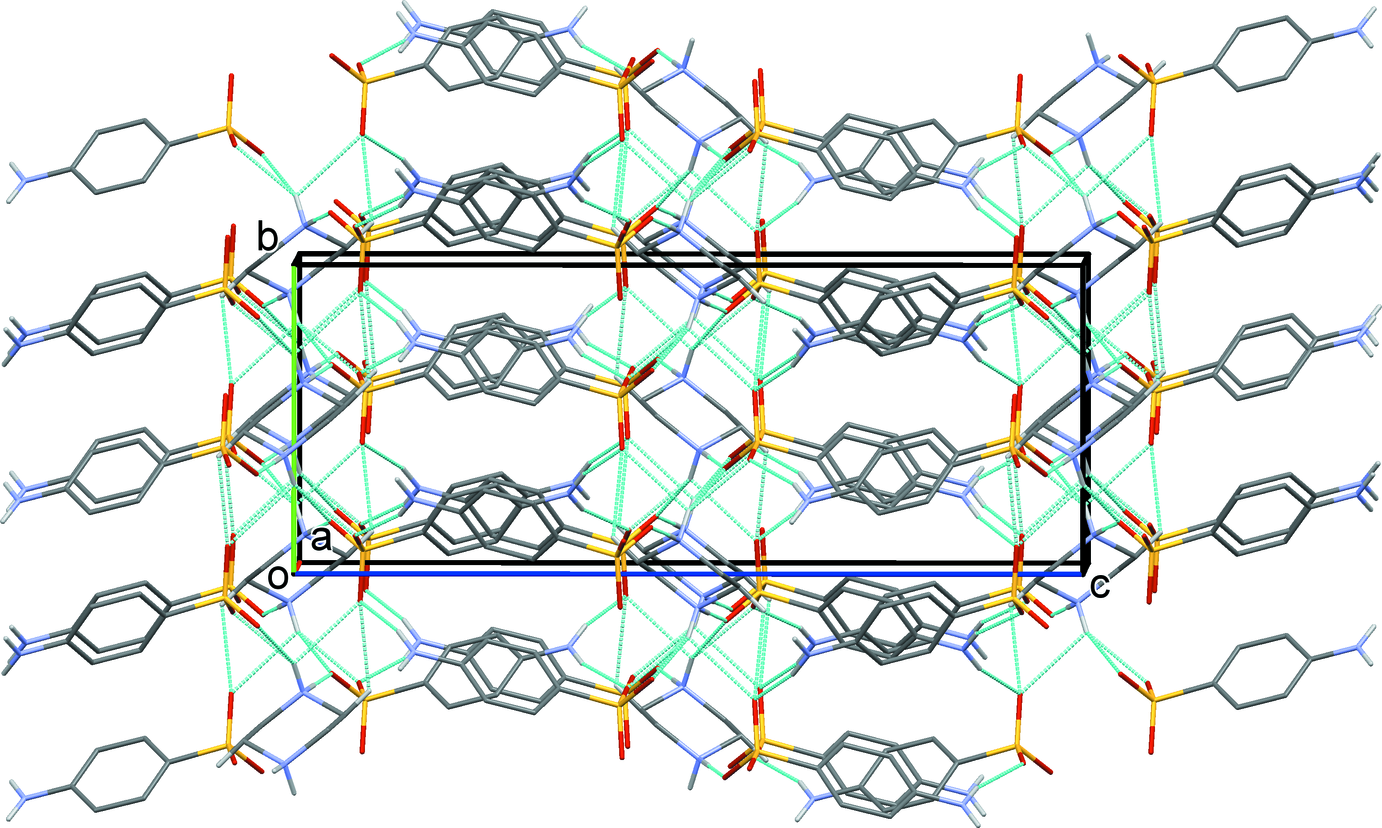

Supplement: Supplementary file 7 [file e-71-o1084-fig4.tif]
